# Bone Mineral Density in Field Hockey Players: A Systematic Review

**DOI:** 10.3390/life14040455

**Published:** 2024-03-29

**Authors:** David Oteo-Gómez, Carlos Castellar-Otín, Alejandro Moreno-Azze, Francisco Pradas de la Fuente

**Affiliations:** 1 Faculty of Health and Sports Sciences, University of Zaragoza, 22001 Huesca, Spain; davidoteo@gmail.com (D.O.-G.); castella@unizar.es (C.C.-O.); franprad@unizar.es (F.P.d.l.F.); 2 ENFYRED Research Group, Faculty of Health and Sports Sciences, University of Zaragoza, 22001 Huesca, Spain

**Keywords:** field hockey, bone density, absorptiometry, sports, sedentary behaviour

## Abstract

The aim of this study was to carry out a systematic review to compare and analyse the bone mineral density of field hockey players of both sexes and of different ages, with other sports and with a sedentary population. The search process was carried out using the PubMed, SPORTDiscus, Web of Science and Scopus databases. The search ended on 18 March 2024. We selected articles in which a comparison was made of bone mineral density of the whole body, lumbar spine, femoral neck, arms and legs, among field hockey players, and/or with other sports and/or with a sedentary population. The systematic review followed the guidelines described in the 2020 PRISMA statement. The initial search identified 220 articles. After applying the inclusion and exclusion criteria, the search was narrowed down to seven articles in total. It was observed that the field hockey group had better bone mineral density values than sedentary population and the low-impact sports population. Basketball players had better whole body and leg bone mineral density values than field hockey players. Causality could not be established due to the cross-sectional nature of the included studies. The better bone mineral density values in field hockey players compared to the sedentary population may be because people who participate in impact sports have a better bone mineral density. The differences in bone mineral density between field hockey and low-impact sports could be related to a lower impact during their practice in these disciplines.

## 1. Introduction

There is a large body of scientific evidence on the potential benefits that people can obtain from regular physical activity (PA) and/or physical exercise (PE). Among others, different aspects can be highlighted such as an increase in maximal oxygen uptake (VO_2max_), a reduction in total body fat and intra-abdominal fat, an improvement in glucose tolerance, a decrease in anxiety and depression, an improvement in physical function and quality of life and a lower probability of suffering osteoporotic fractures [1]. Evidence now supports an inverse relationship between regular PA/PE and osteoporosis [2]. The International Society of Clinical Densitometry defines this skeletal disease as a production of changes made in the microarchitecture of the bone, becoming more porous, caused by a decrease in its bone mineral density (BMD), making it more fragile and susceptible to fractures [1,3]. It can also be defined through a BMD T-score measurement in the lumbar spine, hip or femoral neck with values of <2.5 [4].

Osteoporosis is a disease which causes great economic costs to society [5], and it is therefore of great interest to avoid having low levels of BMD and bone mineral content (BMC). Although these levels are partly genetically determined [6], healthy BMD levels can be achieved by maintaining an appropriate lifestyle, including regular PA and exercise [7], among other measures. Exercise training helps to improve BMD and BMC, increases the internal bone strength of bones under stress, which translates into preventing, delaying or reversing bone loss [8,9]. By achieving a 10% gain in peak BMD in the optimal time period in which the highest BMC levels are reached, it is possible to delay the onset of osteoporosis by up to 13 years [10], this being even more important in women, due to the greater decline in BMC they suffer as a result of menopause [3].

However, even though it is known that PA and PE help to improve bone health, it is still not known what the most effective dose of exercise is to improve BMD [11] or to achieve maximum bone growth [7]. This situation may have its origin in the fact that not all exercises elicit the same osteogenic stimulation. Sports, PA or PE can be divided according to whether they are characterised by high impact, low impact or repetitive [12] or whether they are weight bearing or not. According to Calbet et al. [13] the effect of PE on BMD is observed in sports such as football, which is characterised by being an impact activity, or where weight is supported as two osteogenic activities are present in its nature, such as running and jumping. On the other hand, there are sports or activities which are not related to an improvement in BMD, such as swimming and cycling, as they are non-weight-bearing activities due to their low joint impact [14,15].

Currently, there are numerous systematic reviews and meta-analyses on certain sports, such as football, in which attempts are made to find out how the characteristics of this type of sport influence the BMD of the players and athletes who play it [14,15,16]. However, there are other disciplines, considered to be of less relevance and sporting influence, classified as medium impact, as is the case of field hockey [17], which are of less interest. Scarce scientific evidence is available regarding what effect practicing these sports may have on BMD. Field hockey can be defined as a team sport practised in the open air, in which a great variety of intermittent efforts follow one after the other in a continuous manner. This sport is characterised by a great variability in the intensity of play, with players performing a combination of very high-intensity efforts with other low-intensity movements, as well as periods of inactivity [18]. In this sense, field hockey will require a good physical condition, both anaerobic and aerobic, and great power as a result of the continuous presence of accelerations, decelerations and sprints [19].

Considering the scarce research carried out in the field hockey environment from an osteogenic point of view, the aim of this study is to perform a systematic review to compare and analyse the bone mineral density of field hockey players, of both sexes and of different ages, with other sports and with a sedentary population.

## 2. Materials and Methods

### 2.1. Search Strategy

The PRISMA 2020 guidelines for systematic reviews were followed for this systematic review [20,21,22,23]. This systematic review was registered in PROSPERO, international prospective register of systematic reviews (https://www.crd.york.ac.uk/PROSPERO/ accessed on 16 March 2024) with the code CRD42024510991 prior to implementation. Taking into account the main objective of the study, and in order to carry out a valid search, the PICO question was formulated as follows: (i) the population was men and women of different ages, (ii) the intervention was field hockey, (iii) the comparison groups were field hockey players, players of any other sport, and/or sedentary population, (iv) and the outcomes or variables were BMD of whole body, femoral neck, lumbar spine, arms and legs. We chose the femoral neck and lumbar spine variables because the International Society of Clinical Densitometry uses them to define whether a person has osteoporosis [4], and because they are the sites most susceptible to osteoporotic fractures [24]. BMD in the whole body, arms and legs provides global information on the state of bone health of the subject, and helps to compare between sports, because some disciplines can have more effect on the lower body, on the upper body or even in some cases on both upper and lower limbs.

The search process was carried out in the following databases, the last one on 18 March 2024: PubMed, Scopus, Web of Science and SPORTDiscus, using MeSH terms in all fields. The search strategy in each database was carried out as follows:

- PubMed: (((“Hockey”[Mesh] OR “field hockey”) AND (“Sports”[Mesh] OR “Sports”)) AND (“Absorptiometry, Photon”[Mesh] OR “dual-energy x-ray absorptiometry”)) AND (((“Bone Density”[Mesh] OR “Bone Density”)) OR (“Bone and Bones”[Mesh] OR Bone and Bones)).
- Scopus: (((“Hockey” OR “field hockey”) AND (“Sports”)) AND (“dual-energy x-ray absorptiometry”)) AND ((“Bone Mineral Density”) OR (“Bone and Bones”))
- SPORTDiscus: (((“Hockey” OR “field hockey”) AND (“Sports”)) AND (“dual-energy x- ray absorptiometry”)) AND ((“Bone Mineral Density”) OR (“Bone and Bones”)).
- Web of Science: (ALL = (Hockey) OR ALL = (field hockey)) AND (ALL = (Sports)) AND (ALL = (Absorptiometry, Photon) OR ALL = (dual-energy x-ray absorptiometry)) AND (ALL = (Bone Density) OR ALL = (Bone and Bones)).

### 2.2. Inclusion and Exclusion Criteria

The studies were selected on the basis of the following criteria: (1) use of dual photon X-ray absorptiometry (DXA) to obtain BMD data, (2) studies comparing field hockey players, or with other sports, or with a sedentary population, (3) selected articles are cross-sectional, longitudinal, cohort or randomised controlled trials, (4) research in which at least one of the following variables is analysed: BMD of whole body, femoral neck, lumbar spine, arms or legs.

On the other hand, the exclusion criteria established were the following: (1) systematic reviews, meta-analyses, case studies, case–control studies and letters to the editor, (2) unpublished literature, (3) the participants had underlying medical conditions, (4) publications not written in Spanish or English.

### 2.3. Quality Assessment and Risk of Bias

A standardised instrument was used to check the quality of the studies. The tool used to assess bias in cross-sectional studies was adapted by Lozano et al. [25] from Downs and Black [26]. This tool consists of ten items, the first six items refer to descriptive information, one item refers to external validity, two items refer to internal validity, and the last item refers to clinical effects. If the article complied with any item, it was scored with a value of 1, if it did not, it was scored with a value of 0, and if it could not be applied, the text was incorporated as “Not available”. Subsequently, the score was added up and the percentage was obtained, with the value of 10 being 100%. The protocol proposed by Lozano et al. [13] was also followed for scoring according to the percentage obtained, with the following evaluation method: 0–20% = poor, 21–40% = poor, 41–60% = fair, 61–80% = good, and 81–100% = good.

## 3. Results

### 3.1. Main Search

**Figure 1:**
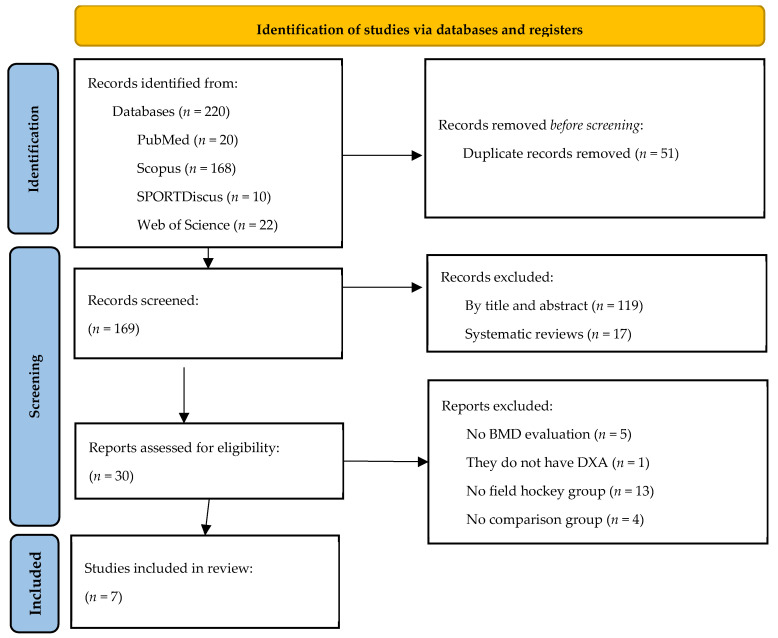
PRISMA flowchart.

The initial search showed 220 articles, of which 20 were from PubMed, 168 from Scopus, 22 from Web of Science and 10 from SPORTDiscus. After reviewing duplicate articles, 51 articles were eliminated, leaving a total of 169 articles in the total review. After applying the inclusion and exclusion criteria, the search was definitively reduced to seven articles, which formed the final process of the systematic review. Figure 1 shows the flowchart specifying the process followed in the selection and filtering of the studies analysed from the initial search.

### 3.2. Quality Assessment and Risk of Bias

Based on the tool adapted from Lozano et al. [25], the following scores were obtained: four articles achieved a score of 80% (good), one study a score of 70% (good), and two articles a score of 50% (fair). Averaging the seven articles resulted in a score of 70%, so the quality of the studies was considered to be good.

Although one of the studies included in the systematic review was of a longitudinal observational nature, it was assessed as cross-sectional, as the data of interest for this systematic review were only assessed on a single occasion. The quality assessment process of the studies, as well as the selection process, was carried out by two researchers, with any disagreement resolved through consensus.

### 3.3. Results of the Studies

The seven articles included in the systematic review are described in Table 1. Six articles were found that included the female sex and only one article had a male population. Four studies compared field hockey with other sports, while one article compared different athletes with different levels of field hockey. Four articles compared field hockey with a sedentary population. Of the studies found, six were cross-sectional observational studies and there was only one longitudinal observational study. On the other hand, the most analysed variables were whole body BMD and BMD in the legs. The results of the systematic review are presented below, grouped into three sections differentiated by the comparison groups of the study.

#### 3.3.1. Comparison between Field Hockey Players and Controls

The first study to assess BMD by comparing field hockey players with sedentary players was the study by Dook et al. [17]. In this investigation, the participants were 46.2 (3.1) year old female veteran players with more than 20 years of sporting experience. It was observed that the female field hockey players had higher BMD values for whole body, arms and legs than their comparison group (*p* < 0.05).

Bellver et al. [24] showed that female field hockey players aged 23.8 (3.7) years had higher BMD of the whole body, femoral neck, lumbar spine, arms and legs than the sedentary group (*p* < 0.05). Similar results were found in the study by Beck et al. [27], where the slightly younger (20 years) female field hockey players showed higher BMD of the whole body (*p* = 0.02), femoral neck (*p* < 0.01) and lumbar spine. However, only in the first two variables were the differences statistically significant.

The only study conducted on male field hockey players aged 21.4 (1.6) years was by Krzykała et al. [30]. They obtained results in which the group of field hockey players had higher values for the whole body, left leg and right leg, but they were significant only for the left leg values (*p* < 0.05). On the other hand, BMD values in both arms were higher in the sedentary group, but the differences were not significant.

#### 3.3.2. Comparison between Field Hockey Players

Krzykała et al. [31] compared the Polish women’s national field hockey team, with a mean age of 21.01 (3.83) years, with a group of junior field hockey players, with a lower sporting level and a mean age of 17.27 (0.85) years. The results show significant differences in favour of the group of female youth players in the BMD of both arms (*p* < 0.05). BMD in the lower extremities was also assessed and in both, the national team players had better values, but these differences were not significant.

#### 3.3.3. Comparison between Field Hockey Players and Other Sports

All studies included in the review that compared a field hockey group with other sports were composed of a female athletes. The research by Dook et al. [17] was also the first to make this comparison, where we found sports such as basketball, netball and swimming. Female field hockey players had significantly better values for leg BMD when compared to swimmers (*p* < 0.05). Basketball and netball players had better values for whole body, arm and leg BMD compared to field hockey players, but these were not statistically significant. Something similar occurs between field hockey players and swimmers in whole body and arm BMD, with better values in the field hockey group, but these differences were not significant.

Bellver et al. [24] carried out a comparison of non-aquatic sports (field hockey, football and volleyball) with aquatic sports (swimming, water polo and synchronised swimming) in athletes aged 19–24 years. The authors found that field hockey players had better values than swimmers for whole body and femoral neck BMD and synchronised swimming athletes for whole body BMD (*p* < 0.05). Volleyball and football players had better values for whole body, femoral neck, lumbar spine and leg BMD than field hockey players, but the differences were not significant. On the other hand, water polo players had better BMD values for femoral neck, lumbar spine, arms and legs than field hockey players but the differences were not significant.

Dobrosielski et al. [28] performed a comparison of whole body, arm and leg BMD in 12 different sports with athletes aged 19 years. Female field hockey players showed significant differences in their favour in whole body BMD when compared to those in swimming, diving and cross country (*p* < 0.01). The same occurs when the variable is BMD in arms but with the sports of athletics (track) and cross country and with the variable BMD in legs when compared with athletes in swimming and diving (*p* < 0.01). In addition, basketball players have higher values in their favour when compared to field hockey players in whole body and legs (*p* < 0.01). Similarly, athletics (throwing) have better values for arm BMD than field hockey players (*p* < 0.01).

Finally, the study by Mudd et al. [29] which aimed to compare whole body, lumbar spine and leg BMD in eight sports with 20-year-old female athletes, observed that female field hockey players showed higher values of whole body and leg BMD when compared to swimming and diving (*p* < 0.01). On the other hand, there were no significant differences in the variable lumbar spine BMD between field hockey players and players of other sports.

## 4. Discussion

### 4.1. Interpretation of the Results

The objective of this study has been to carry out a systematic review in which the bone mineral density of field hockey players, of both sexes and of different ages, is analysed with other sports and/or with a sedentary population. Thanks to the search strategy and the established inclusion and exclusion criteria, it was possible to compare five BMD variables (whole body, femoral neck, lumbar spine, arms and legs). The discussion will be presented in three sections, following the same order of presentation of the results and differentiated according to the comparison groups of the study.

### 4.2. Comparison between Field Hockey Players and Controls

In the three studies which compared the female athletes, it can be observed that the female field hockey players obtained better BMD values in all the variables analysed. This may be due to the continuous practice of these athletes in the sport of field hockey for several years, since as can be seen in the different studies analysed, the shortest period of practice by the female players is seven years [27]. When practising an impact sport that involves continuous movement, as is the case with field hockey, the bone tends to adapt to the loads it suffers, improving its quality and resistance thanks to the processes of bone modelling and remodelling, which is known as Frost’s mechanostat theory [32]. In this sense, if sportswomen have practised sport during their growth stage, they can increase the maximum BMD of those bones which undergo loading by between 10% and 20% [33]. The effects of this increase in BMD during growth have been analysed in the study by Dook et al. [17], carried out on women athletes aged 46.2 (3.1) years, where it was found that the effects of playing field hockey for more than 20 years on BMD are maintained over time, possibly related to improved BMD. The practice of sports, such as field hockey, during growth and throughout much of adult life appears to be associated with adequate BMD values as bone and vital ageing progress. However, Sparling [34] comments that although there are differences in favour of elite female athletes in terms of BMD, and while these differences are consistent, they are considered to be relatively small.

On the other hand, one study [35] found that each additional hour of sedentary behaviour was associated with a BMD femoral neck of −0.006 g/cm^2^ in children, which translates into a 0.7% loss of BMD femoral neck for each hour of sedentary behaviour [35]. This means that sedentary people not only fail to improve their bone mass, but simultaneously worsen it. However, female field hockey players, thanks to the stress on their bones, improve their bone quality and strength. Similar results have been found in studies of sports which have BMD values similar to field hockey, such as football, where female football players and sedentary controls were compared, with the female football players obtaining better BMD results [25].

However, when the male sex is analysed, the same results are not found. Significant differences have only been obtained in BMD of the left leg [30]. On the contrary, in studies related to football, differences were found in several variables when the same comparison was made [25,36]. The reason why so many differences are not observed may be due to the control group, because the authors do not describe the characteristics of this group adequately, so it may not be the ideal group to be able to carry out this type of comparison.

### 4.3. Comparison between Field Hockey Players

In the only study comparing two groups of female field hockey players, it has been observed that the group of junior players showed differences in their favour in the upper extremities. Due to the scarcity of existing studies in the scientific literature, insufficient studies have been found to be able to compare and justify why junior players show better BMD values in the upper extremities, and similar results in the lower extremities, without being high performance players, and having fewer years of sporting experience (10 years vs. 5 years). An important aspect relating to the characteristics of the sample, and which has not been carried out in any of the studies analysed, is that there is no mention of whether these players competed in the indoor hockey modality. It is very common that during the winter break of the hockey season, especially in northern European countries where the sport is more popular, players who are not committed to the national team (due to the fact that these periods of time are used for international competitions), compete in indoor hockey. Indoor hockey is played on a harder surface and not on artificial turf like hockey. The practice of indoor hockey could be related to a greater osteogenic stimulation as a consequence of the playing field where it is played [25]. In this sense, Lozano et al. [25] insist on this aspect in their study when comparing futsal with football played on artificial turf.

On the other hand, it is interesting to note that the authors of the study [25] reported that among the senior players of the national team there are significant differences in the lower limbs in favour of the left side. The same differences have also been found in junior players, although they were not statistically significant. These differences were also found in male athletes. Krzykała et al. [30], mention that large differences are observed on the left side of the lower extremities in hockey players, due to a feature in the nature of the sport itself. Players tend to perform very regularly a rotational movement from the right to the left side at high speed, causing a higher mechanical load during play on the mentioned body segment, resulting in higher BMD values. In another study by the same author [37], better BMD results were also obtained for the left leg and trunk than for the right-sided segments. Taking into account these results, as this author comments in both investigations, hockey is an asymmetrical sport, also due to the fact that differences are not only found between the right and left side in BMD but also in muscle mass [37].

### 4.4. Comparison between Field Hockey Players and Other Sports

The BMD value of the whole body of the field hockey players who belonged to the US Olympic team was one of the highest in comparison with other female team sports (1.253 g/cm^2^) [34]. However, as can be seen in the results of our review, there are sports that have obtained better values in some of the variables analysed, such as basketball. In a study where different variables were analysed in National Basketball Association (NBA) teams, data were found on the reaction forces that these players suffered during some games. The reaction forces were up to 6 times body weight in gestures such as the jump shot, or 4.3 times body weight in landings after a vertical jump [38].

No studies have been found that show the same data in field hockey, but in actions that are present in field hockey, such as running, which generate forces of 1.6 to 3 times body weight [39]. Jumping actions are very present within the sport of basketball [38], and are one of the most osteogenic exercises that exist, on the contrary, jumps in field hockey are not repeated as frequently and with the same intensity, which is why better BMD values of whole body and legs are shown in basketball players.

The same reasoning can be followed to explain why field hockey has better BMD values than other sports such as swimming, cross country, scuba diving and synchronised swimming (in a young population). Similar results were observed in other studies comparing swimming with other sports of higher impact and load: swimming had worse BMD values for whole body, femoral neck and lumbar spine [15]. These so-called low-impact sports have a low bone mass because, as the name suggests, they lack the necessary impact [14], which allows the bone to gain strength and quality. For a sport to effectively stimulate osteogenesis, certain factors are necessary to characterise the mechanical loads on the bones, such as the magnitude of stress, frequency and speed [7,40]. Swimming, diving and other similar sports have a low or no impact magnitude, which cannot be compensated for by having a higher impact frequency or velocity, whereas field hockey has a higher impact magnitude. In this sense, better BMD values have been observed in this sport. However, if we analyse the results of the study obtained in players aged 46.2 (3.1) years [17], the differences between field hockey and swimming are reduced, and are only significant in the legs (segments that suffer more mechanical load in field hockey).

However, some limitations of this systematic review are acknowledged. Currently in the scientific evidence, there are few articles that deal with the subject of field hockey and BMD, so the number of articles that have formed part of the review have been reduced. This problem also hinders the subsequent comparison made in the discussion. Also, all the articles included did not have a representative sample of the population, as their sample size was small and their power was not sufficient to detect a clinically significant effect. On the other hand, there is no mention in the articles of any strategies for blind or double-blind treatments, and in some studies there was no control group (which would be interesting if they were not physically active). In addition, there was a linguistic bias in choosing only articles that were written in Spanish or English.

In this systematic review, there is also strengths that can be summarised as follows: (1) An adequate methodology has been developed due to the fact that the guidelines of the PRISMA 2020 Declaration for Systematic Reviews have been followed. (2) The search and selection process of the articles was carried out in accordance with the established inclusion and exclusion criteria. (3) A comparison of field hockey with different types of sports (high impact, low impact, non-weight bearing and weight bearing), with sedentary population and among players of the same sport has been carried out.

## 5. Conclusions

Field hockey players (mainly female) have a lower risk of suffering osteoporosis or osteopenia than the sedentary population or athletes practising low-impact sports.

Field hockey is not one of the sports of greatest interest for obtaining better BMD and bone health values, as it is classified as a medium-impact sport. However, it is a sport that seems to stimulate the osteogenic effect in an effective way, so it could be recommended to the general population.

The sport of field hockey can be considered as a sport discipline that does not need an additional impact intervention, outside the training sessions of the sport itself, to improve the bone health of its players, as other low-impact sports, such as swimming or cycling, do.

## Figures and Tables

**Table 1:** Description of the studies included.

Authors and Year	Study Design	Sample + Level	Gender and Age	Variable of Interest and Measurement	BMD Results	
Bellver et al. [24] Year 2019	Cross-sectional observational	Field hockey *n* = 29 Level: >=3 years practicing elite sport	F: 23.8 ± 3.7	BMD (g/cm^2^) as measured by DXA in whole body, femur neck, lumbar spine, arms and legs	Field hockey Whole body: 1.222 ± 0.1 Femoral neck: 1.155 ± 0.11 Lumbar spine: 1.258 ± 0.10 Arms: 0.701 ± 0.04 Legs: 1.230 ± 0.09	Whole body BMD values in field hockey significantly (*p* < 0.05) + swimming, synchronised swimming and control group. BMD values of femoral neck in field hockey significantly (*p* < 0.05) + swimming and control group. Lum-bar spine BMD values in field hockey significantly (*p* < 0.05) + control group. BMD values of arms in field hockey significantly (*p* < 0.05) + control group. BMD values of leg BMD in field hockey significantly (*p* < 0.05) + control group. BMD values in whole body, femur neck, lumbar spine, arms and legs with respect to field hockey, volleyball, water polo and football there are no significant differences.
		Swimming *n =* 19 Level: >3 years practising elite sport	F: 18.4 ± 3.6		Swimming Whole body: 1.090 ± 0.1 Femoral neck: 0.994 ± 0.10 Lumbar spine: 1.161 ± 0.14 Arms: 0.704 ± 0.05 Legs: 1.109 ± 0.08	
		Water polo *n =* 14 Level: >3 years practising elite sport	F: 24.0 ± 3.7		Water polo Whole body: 1.206 ± 0.1 Femoral neck: 1.172 ± 0.12 Lumbar spine: 1.265 ± 0.09 Arms: 0.894 ± 0.13 Legs: 1.346 ± 0.25	
		Synchronised swimming *n =* 24 Level: >3 years practising elite sport	F: 20.5 ± 3.9		Synchronised swimming Whole body: 1.068 ± 0.1 Femoral neck: 1.103 ± 0.09 Lumbar spine: 1.107 ± 0.11 Arms: 0.697 ± 0.05 Legs: 1.081 ± 0.10	
		Football *n =* 92 Level: >3 years practising elite sport	F: 22.0 ± 5.2		Football Whole body: 1.262 ± 0.1 Femoral neck: 1.240 ± 0.14 Lumbar spine: 1.341 ± 0.16 Arms: 0.697 ± 0.05 Legs: 1.346 ± 0.10	
		Volleyball *n =* 26 Level: >3 years practising elite sport	F: 22.5 ± 4.5		Volleyball Whole body: 1.279 ± 0.1 Femoral neck: 1.272 ± 0.14 Lumbar spine: 1.431 ± 0.18 Arms: 0.743 ± 0.04 Legs: 1.345 ± 0.14	
		Sedentary (control group) *n =* 126	F: 21.5 ± 4.6		Sedentary Whole body: 0.943 ± 0.2 Femur neck: 0.903 ± 0.14 Lumbar spine: 1.057 ± 0.16 Arms: 0.659 ± 0.07 Legs: 1.108 ± 0.12	
Beck et al. [27] Year 2005	Longitudinal observational	Field hockey *n* = 15 Level: not available 7.7 years practising field hockey	F: 20.6 (1.1)	BMD (g/cm^2^) measured by DXA in femoral neck, lumbar spine and whole body	Whole body BMD values in field hockey significantly (*p* = 0.02) + control group. Femoral neck BMD values in field hockey significantly (*p* = 0.00004) + control group. In lumbar spine there is no significant difference.	
		Sedentary (control group) *n =* 17	F: 19.5 (1.5)			
Dobrosielski et al. [28] Year 2021	Cross-sectional Observational	Field hockey *n =* 35 Level: NCAA Division I	F: 18.8 (1.0)	BMD (g/cm^2^) measured by DXA on whole body, arms and legs	Field hockey Whole body: 1.29 (0.08) Arms: 0.96 (0.67) Legs: 1.34 (0.10)	Whole body BMD values in field hockey significantly (*p* < 0.01) + cross country and swimming and scuba diving. BMD values of arms in field hockey significantly (*p* < 0.01) + cross country and track and field (running). BMD values of legs in field hockey significantly (*p* < 0.01) + swimming and scuba diving Whole body BMD values in basketball significantly (*p* < 0.01) + field hockey. BMD values of legs in basketball significantly (*p* < 0.01) + field hockey. Arm BMD values in track and field (throwing) significantly (*p* < 0.01) + field hockey.
		Basketball *n =* 28 Level: NCAA Division I	F: 19.4 (1.3)		Basketball Whole body: 1.40 (0.11) Arms: 1.02 (0.07) Legs: 1.47 (0.12)	
		Cross country (cycling) *n =* 11 Level: NCAA Division I	F:19.7 (1.2)		Cross country Whole body: 1.17 (0.07) Arms: 0.83 (0.78) Legs: 1.23 (0.08)	
		Gymnastics *n =* 23 Level: NCAA Division I	F:19.0 (1.0)		Gymnastics Whole body: 1.31 (0.07) Arms: 1.02 (0.10) Legs: 1.34 (0.08)	
		Lacrosse *n =* 48 Level: NCAA Division I	F: 19.2 (1.2)		Lacrosse Whole body: 1.30 (0.09) Arms: 0.98 (0.07) Legs: 1.36 (0.10)	
		Football *n =* 27 Level: NCAA Division I	F: 19.4 (0.8)		Football Whole body: 1.32 (0.08) Arms:0.94 (0.09) Legs: 1.39 (0.10)	
		Softball *n =* 24 Level: NCAA Division I	F: 19.2 (1.2)		Softball Whole body: 1.35 (0.09) Arms: 1.01 (0.06) Legs: 1.40 (0.09)	BMD values in the whole body, arms and legs comparing field hockey and the other sports there are no significant differences.
		Swimming and scuba diving *n =* 35 Level: NCAA Division I	F:19.5 (1.1)		Swimming and scuba diving Whole body: 1.21 (0.06) Arms: 0.94 (0.07) Legs: 1.21 (0.07)	
		Tennis *n =* 11 Level: NCAA Division I	F: 18.6 (0.7)		Tennis Whole body: 1.26 (0.09) Arms: 0.92 (0.11) Legs: 1.21 (0.07)	
		Track and field (throwing) *n =* 10 Level: NCAA Division I	F: 19.7 (1.5)		Track and field (throwing) Whole body: 1.43 (0.12) Arms: 1.08 (0.07) Legs: 1.49 (0.15)	
		Track and field (running) *n* = 10 Level: NCAA Division I	F: 18.4 (0.5)		Track and field (running) Whole body: 1.22 (0.09) Arms: 0.87 (0.05) Legs: 1.31 (0.13)	
		Volleyball *n* = 16 Level: NCAA Division I	F: 19.6 (1.5)		Volleyball Whole body: 1.36 (0.10) Arms: 1.02 (0.09) Legs: 1.40 (0.11)	
Dook et al. [17] Year 1997	Cross-sectional observational	High impact (basketball and netball) *n* = 20 Level: not available. >20 years of sports practice	F: 45.5 (3.1)	BMD (g/cm^2^) measured by DXA on whole body, arms and legs	Basketball and netball Whole body: 1.15 (0.08) Arms: 0.73 (0.05) Legs: 1.20 (0.09)	Whole body BMD values in field hockey significantly (*p* < 0.05) + control group. BMD values of arms in field hockey significantly (*p* < 0.05) + control group. BMD values of legs in field hockey significantly (*p* < 0.05) + swimming and control group.
		Medium impact (field hockey) *n* = 20 Level: not available. >20 years of sports practice	F: 46.2 (3.1)		Field Hockey and running Whole body: 1.12 (0.10) Arms: 0.71 (0.05) Legs: 1.18 (0.09)	
		No impact (swimming) *n =* 20 Level: not available. >20 years of sports practice	46.0 (3.6)		Swimming Whole body: 1.06 (0.08) Arms: 0.71 (0.05) Legs 1.11 (0.09)	
		Sedentary (control group) *n* = 20	45.6 (2.1)		Sedentary Whole body: 1.02 (0.07) Arms: 0.67 (0.05) Legs: 1.05 (0.08)	
Mudd et al. [29] Year 2007	Cross-sectional observational	Field hockey *n* = 10 Level: NCAA Division I	F: 19.8 ± 1.2	BMD (g/cm^2^) measured by DXA in lumbar spine, whole body and legs	Field hockey Whole body: 1.161 ± 0.095 Lumbar spine: 1.311 ± 0.120 Legs: 1.268 ± 0.138	Whole body BMD values in field hockey significantly (*p* < 0.01) + swimming and scuba diving. Leg BMD values in field hockey significantly (*p* < 0.01) + swimming and scuba diving. Lumbar spine BMD there are no significant differences between field hockey and other sports.
		Gymnastics *n =* 8 Level: NCAA Division I	F: 19.7 ± 0.9		Gymnastics Whole body: 1.173 ± 0.036 Lumbar spine: 1.213 ± 0.121 Legs: 1.261 ± 0.063	
		Softball *n* = 14 Level: NCAA Division I	F: 20.1 ± 1.1		Softball Whole body: 1.163 ± 0.061 Lumbar spine: 1.171 ± 0.10 Legs: 1.267 ± 0.075	
		Running *n =* 25 Level: NCAA Division I	F: 20.4 ± 1.3		Running Whole body: 1.079 ± 0.055 Lumbar spine: 0.988 ± 0.118 Legs: 1.184 ± 0.072	
		Track and field *n =* 8 Level: NCAA Division I	F: 20.1 ± 1.3		Track and field Whole body: 1.152 ± 0.062 Lumbar spine: 1.104± 0.098 Legs: 1.272 ± 0.098	
		Swimming and scuba diving *n =* 9 Level: NCAA Division I	F: 20.4 ± 1.1		Swimming and scuba diving Whole body: 1.083 ± 0.050 Lumbar spine: 1.079 ± 0.107 Legs: 1.117± 0.086	
		Football *n* = 10 Level: NCAA Division I	F: 19.8 ± 0.9		Football Whole body: 1.149 ± 0.043 Lumbar spine: 1.054 ± 0.108 Legs: 1.276 ± 0.045	
		Rowing: 15 Level: NCAA Division I	F: 20.5 ± 2.1		Rowing Whole body: 1.126 ± 0.063 Lumbar spine: 1.078 ± 0.061 Legs: 1.208 ± 0.076	
Krzykała et al. [30] Year 2018	Cross-sectional observational	Field hockey *n* = 15	M: 21.4 (1.6)	BMD (g/cm^2^) measured by DXA on whole body, arms and legs	Field hockey Whole body: 1.34 (0.1) Left arm: 1.009 (0.09) Right arm: 1.020 (0.09)	BMD values of whole body, arms and right legs in field hockey are not significantly (*p* = 0.08) + control group
		Level: Poland Youth National Team. 10.8 years of sporting practice			Left leg: 1.576 (0.13) Right leg: 1.553 (0.14)	BMD values of left leg in field hockey significantly (*p* < 0.05) + control group
		Control group *n =* 14	M: 22.3 (2.1)		Sedentary Whole body: 1.27 (0.1) Left arm: 1.025 (0.07) Right arm: 1.045 (0.09) Left leg: 1.480 (0.54) Right leg: 1.450 (0.12)	
Krzykała et al. [31] Year 2015	Cross-sectional observational	Poland National Team Field Hockey *n* = 17 Level: National Team. 10 years of sports practice	F: 21.01 (3.83)	BMD (g/cm^2^) measured by DXA in arms and legs	Poland National Team Field Hockey Left arm: 0.86 (0.046) Right arm: 0.87 (0.042) Left leg: 1.39 (0.72) Right leg: 1.36 (0.065)	The field hockey youth team has BMD values in both arms significantly (*p* < 0.05) + national team. In both legs there are no significant differences. The senior field hockey team has BMD values in the left leg significantly (*p* < 0.013) + right leg.
		Youth Team Field Hockey *n =* 14 Level: not available. 5.39 years of sports practice	F: 17.27 (0.85)		Poland National Team Field Hockey Left arm: 0.98 (0.079) Right arm: 1.01 (0.060) Left leg: 1.35 (0.088) Right leg: 1.33 (0.077)	

Note: +: upper, greater than, BMD: bone mineral density, DXA: dual photon X-ray absorptiometry, F: female gender, M: male, *n*: sample population, NCAA: National Collegiate Athletic Association.
